# Association of interleukin-10 promoter polymorphisms and corresponding plasma levels with susceptibility to laryngeal squamous cell carcinoma

**DOI:** 10.3892/ol.2014.1914

**Published:** 2014-02-26

**Authors:** JIAN ZHOU, DUO ZHANG, BIN CHEN, QING LI, LIN ZHOU, FEI LIU, KUANG-YEN CHOU, LEI TAO, LI-MING LU

**Affiliations:** 1 Department of Otolaryngology, Eye, Ear, Nose and Throat Hospital, Fudan University, Shanghai 200031, P.R. China; 2 Shanghai Institute of Immunology, Shanghai Jiaotong University School of Medicine, Shanghai 200025, P.R. China

**Keywords:** laryngeal squamous cell carcinoma, vocal leukoplakia, cytokines, polymorphism, interleukin-10

## Abstract

Interleukin (IL)-10 is critically involved in tumorigenesis. In the present study, the association between the IL-10 −1082/−819/−592 promoter polymorphisms, the plasma IL-10 levels and the risk of laryngeal squamous cell carcinoma (LSCC) was investigated in a prospective, case-control study. In total, 146 patients with LSCC, 61 with vocal leukoplakia and 119 healthy controls were genotyped for the IL-10 gene (IL-10 −1082 A/G, −819 T/C and −592 A/C) using pyrosequencing, and their plasma IL-10 levels were analyzed by ELISA. The patients with LSCC had a significantly higher frequency of AC at position −592 and −819 (OR, 1.82 and P=0.024) compared with the control, and a higher frequency of AG at position −1082 (OR, 2.20 and P=0.037). The patients with advanced LSCC had a significantly higher frequency of AG+GG at position −1082 compared with those with early-stage LSCC (OR, 3.13 and P=0.008 vs. OR, 2.06 and P=0.068). The patients with lymph node metastasis had a significantly higher frequency of AG+GG at position −1082 compared with the patients with no lymph node metastasis (OR, 2.97 and P=0.048 vs. OR, 2.23 and P=0.035). In addition, the patients with high frequencies of each genotype polymorphism had high plasma IL-10 concentrations. The present study indicates that the IL-10 −1082/−819/−592 promoter polymorphisms and corresponding high plasma IL-10 concentrations are associated with LSCC, and that variations in genotype distribution and plasma IL-10 concentrations may be associated with the stage and the lymph node metastasis status of LSCC.

## Introduction

Laryngeal squamous cell carcinoma (LSCC) is the most frequent type of head and neck cancer, the risk of which results from complex interactions between numerous genetic and environmental factors (1). Effective treatments include radiotherapy and chemotherapy, although surgery is currently the only treatment that consistently prolongs survival time (2). The most effective approaches to achieving an improved prognosis in LSCC patients are prevention and early diagnosis. Accumulating evidence indicates that genetic polymorphisms are associated with laryngeal carcinoma, and extensive investigations have been conducted to identify inherited genetic risks for this disease (3).

Vocal leukoplakia is a mucosal epithelial hyperplastic keratosis lesion of the vocal cords and is often considered to be a pre-cancerous lesion of laryngeal carcinoma. Interleukin (IL)-10 is an anti-inflammatory cytokine, which is involved in suppressing T-helper (Th)1 lymphocytes and stimulating B lymphocytes and Th2 lymphocytes to mediate immune responses, downregulating the production of pro-inflammatory mediators, including IL-1β, tumor necrosis factor-α, interferon-γ and other pro-inflammatory cytokines (4–6), and suppressing the antigen presentation capacity of antigen-presenting cells (7). The gene encoding IL-10 is located on chromosome 1 (1q31–1q32) and consists of five exons and four introns.

Numerous polymorphisms of the IL-10 gene promoter have been identified; primarily single nucleotide polymorphisms (SNPs), with three polymorphisms in the 5-flanking region of IL-10 at positions −1082 (A-G), −819 (T-C) and −592 (A-C) (8). These polymorphisms are in strong linkage disequilibrium and are associated with various serum levels of IL-10 *in vivo*. The GCC haplotypes are associated with a high IL-10 production in peripheral blood cell cultures, while the ATA haplotypes are associated with low levels of IL-10 (9,10). Furthermore, numerous studies have indicated that environmental factors, including cigarette smoking and alcohol consumption, induce cytokine expression and are associated with certain cytokine genotypes (11). Cigarette smoking and alcohol consumption may act independently or in synergy with cytokine genotypes to confer individual susceptibility to developing LSCC.

In previous years, several studies have indicated that the IL-10 promoter polymorphisms are associated with an increased risk of cancers, including lung, thyroid, prostate, cervical and gastric cancers (12–16). Nevertheless, studies concerning the interaction of cytokine genes and environmental factors in LSCC are scarce.

In the present case-control association study, the effects of three polymorphisms of IL-10 and their allele frequencies on disease susceptibility and the severity of LSCC were investigated. The interaction between these cytokine genotypes and plasma IL-10 levels, and the association with environmental risks, including cigarette smoking and alcohol consumption, was further investigated.

## Material and methods

### Ethical approval

The study protocol was approved by the Medical Research Council of the Eye, Ear, Nose and Throat Hospital, Fudan University (Shanghai, China; no. KJ2008-01). Informed consent was obtained from each patient and healthy (control) individual.

### Patients and controls

Between October 2012 and February 2013, 146 patients with LSCC and 61 with vocal leukoplakia were enrolled in the study at the Eye, Ear, Nose and Throat Hospital. All patients were of Chinese Han origin and were recruited from various geographical regions of China. Healthy volunteers of equivalent ethnicity, gender and age were enrolled as the control group (n=119) in the study. Informed consent was obtained according to the Declaration of Helsinki. The clinicopathological findings of the cancer group were collected. All the pathological cell types in the cancer group were squamous cell carcinoma. Smoking habits were defined as non-smoker (<100 cigarettes in their lifetime) and smoker (>20 cigarettes per day for ≥1 year), and alcohol consumption was defined as non-drinker and drinker (>200 ml per day). The characteristics of various subgroups are shown in Table I.

### Blood collection and DNA and plasma extraction

Blood (5 ml) was collected from each participant in an EDTA tube and centrifuged for 10 min at 900 × g. Plasma was isolated from peripheral blood and stored at −80°C within 30 min of collection. Genomic DNA (100 ng/μl) was prepared from peripheral blood using a TIANamp Blood DNA kit [Tiangen Biotech (Beijing), Co., Inc., Beijing, China].

### Analysis of plasma IL-10 levels and IL-10 −1082/−819/−592 polymorphisms

The plasma IL-10 levels were analyzed using a standard enzyme-linked immunosorbent assay (ELISA); Human IL-10 Platinum ELISA (74540061; eBioscience, San Diego, CA, USA).

The IL-10 (−592, −819 and −1082) polymorphisms were analyzed by PCR amplification of the promoter or coding regions using specifically designed pairs of oligonucleotide primers followed by direct sequencing (ABI Prism 3730xl DNA sequencer; PE Applied Biosystems, Foster City, CA, USA). For the IL-10 genotyping, the PCR conditions were as follows: 30 cycles of 98°C for 10 sec, 55°C for 15 sec and 72°C for 1 min. All the laboratory assays were conducted and interpreted blindly without any knowledge of the case or control status. The primer sequences (17) used in the present study were: Sense, 5′-ATCCAAGACAACACTACTAA-3′ and antisense, 5′-TAAATATCCTCAAAGTTCC-3′; and direct sequencing with primer, 5′-TAAATATCCTCAAAGTTCC-3′.

### Statistical analysis

The demographic characteristics and environmental factors, and the gene frequencies of IL-10 in patients and controls were compared and tested using χ^2^ tests. Since environmental variables, including cigarette smoking and alcohol consumption, were the main risk factor for LSCC in the present study, these factors were also dichotomized and their effects on the risk of LSCC were investigated. Logistic regression analyses were used to evaluate the effects of genotypes, plasma IL-10 and cigarette smoking.

The data were analyzed using the SPSS statistical package (SPSS, Inc., Chicago, IL, USA). Odds ratios (ORs) and 95% confidence intervals (CIs) are presented. P<0.05 was considered to indicate a statistically significant difference.

## Results

### Demographic details

The characteristics and risk factors in the patients with LSCC and vocal leukoplakia and in the controls are presented in Table I. There was no statistical difference between the ages of the controls (62.32±7.9 years), the LSCC patients (60.91±8.7 years) and the vocal leukoplakia patients (56.54±10.7 years). However, smoking was a risk factor of LSCC (OR, 6.33; 95% CI, 3.7–10.8; P<0.01) and vocal leukoplakia (OR, 4.73; 95% CI, 2.4–9.1; P<0.01). Alcohol consumption was also a risk factor of LSCC (OR, 5.67; 95% CI, 3.3–9.9; P<0.01) and vocal leukoplakia (OR, 5.70; 95% CI, 2.9–11.2; P<0.01). The type of carcinoma, stage of LSCC and lymph node metastasis status are also presented in Table I.

### Genotype and haplotype frequency distribution between controls and cases

The distribution of the cytokine gene polymorphisms among the subjects with LSCC and vocal leukoplakia and the controls is summarized in Table II. The results show that the risk for LSCC is associated with the IL-10 polymorphism. The IL-10 genotype containing the G allele at position −1082 or the C allele at positions −819 or −592 was more frequent in cases of LSCC or vocal leukoplakia when compared with the controls (Table II). Unexpectedly, it was observed that the genotypes at position −592 were changed synchronously with that of −819 in the patients. A 1.82-fold increased susceptibility to LSCC was observed with the presence of AC at IL-10 −592 and −819 (P=0.024), while the OR for vocal leukoplakia was 1.93 (P=0.050). Compared with individuals with the AA genotype, the relative risk (OR) of the development of LSCC for heterozygotes with AG at position −1082 was 2.20 (95% CI, 1.04–4.67; P=0.037; Table II) and for vocal leukoplakia this was 2.14 (95% CI, 0.87–5.27; P=0.092; Table II). However, the OR of the CC genotype at positions −592 and −819 in LSCC was 0.83 (CI, 0.37–1.86; P=0.642) and for vocal leukoplakia this was 1.91 (CI, 0.78–4.72; P=0.164).

### Genotype and haplotype frequency distribution based on stages of cancer

For the genotypic comparison of the patients with differing stages of cancer, individuals were categorized into three groups: Control, early (stages I and II) and advanced (stages III and IV). The present results did not show any risk for LSCC associated with CC polymorphisms at IL-10 −592 and −819. With regard to the IL-10 −1082 polymorphism, a 1.80-fold increased risk for early-stage LSCC associated with AC at −592 and −819 (P=0.046) and a 1.88-fold increased risk for advanced LSCC (P=0.080) was observed. A 2.06-fold increased susceptibility for early-stage LSCC associated with the G allele (GA/GG) at IL-10 −1082 (P=0.068) was also observed, while for advanced LSCC the OR was 3.13 (P=0.008). Therefore, patients with advanced LSCC had a significantly higher OR when compared with those with early-stage LSCC (Table III).

### Genotype and haplotype frequency distribution based on lymph node metastasis

The association between the IL-10 −592, −819 and −1082 gene variants and the lymph node metastasis status was also determined. No significant association for metastasis risk with the IL-10 −592 and −819 polymorphisms was observed. However, the OR for patients with lymph node metastasis at −1082 GA/GG was 2.97 (P=0.048), and 2.23 (P=0.035) (Table IV) in the no lymph node metastasis group. These data indicated that the patients with the IL-10 −1082 GA/GG genotypes were at an increased risk of lymph node metastasis.

### Plasma IL-10 concentrations in controls and cases

**Figure 1:**
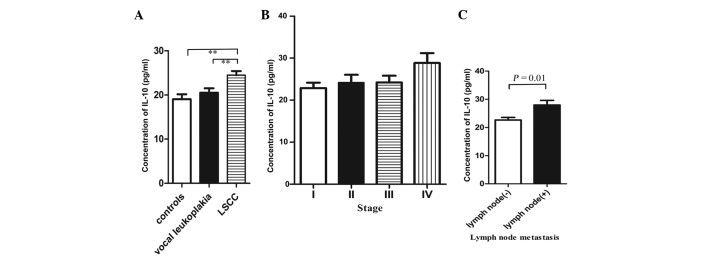
(A) Plasma IL-10 concentrations in controls and cases. IL-10 concentrations were analyzed using a standard ELISA and the data were analyzed using the SPSS statistical package; ^**^P<0.01. (B) Concentration of plasma IL-10 at different cancer stages. (C) Concentration of plasma IL-10 in patients with and without lymph node metastasis. LSSC, laryngeal squamous cell carcinoma; IL-10, interleukin-10; ELISA, enzyme-linked immunosorbent assay.

Variations in plasma IL-10 concentrations were observed in the patients with LSCC and vocal leukoplakia and in the control group (Fig. 1A). In the LSCC patients, the plasma IL-10 concentrations (24.47±5.4 pg/ml) were significantly higher compared with those in the controls (19.02±7.01 pg/ml; P<0.01) and the vocal leukoplakia patients (20.33±3.1 pg/ml; P=0.001), although the concentrations in the vocal leukoplakia patients were also higher than those in the controls.

### Association of plasma IL-10 concentrations and stages of cancer and lymph node metastasis

The variations in plasma IL-10 concentrations were observed in the patients with differing stages of cancer (Fig. 1B). The concentration of plasma IL-10 was found to increase with the cancer staging. The concentration of plasma IL-10 at stage IV (28.84±5.8 pg/ml) was significantly higher than at other stages (I: 22.88±4.8 pg/ml, P=0.028; II: 24.19±6.1 pg/ml, P=0.121; III: 24.23±4.8 pg/ml, P=0.051). The concentrations of plasma IL-10 in the patients with lymph node metastasis (27.95±5.7 pg/ml) were significantly higher compared with the patients with no lymph node metastasis (22.66±4.4 pg/ml) (P=0.01; Fig. 1C).

### Plasma concentration of IL-10 in association with IL-10 genotype polymorphism

**Figure 2:**
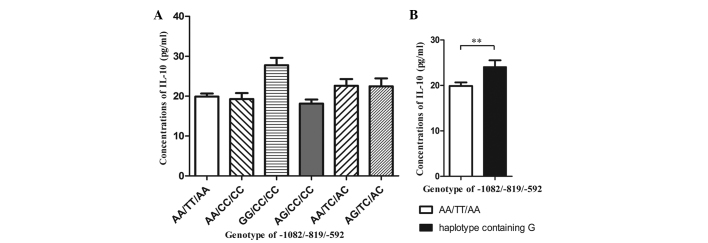
(A) Plasma IL-10 concentrations in the different IL-10 genotypes. (B) Plasma IL-10 concentrations in patients with the AA/TT/AA genotype and patients with haplotypes containing G (GG/CC/CC; AG/CC/CC; and AG/TC/AC); ^**^P<0.01. IL-10, interleukin-10.

Variations in plasma IL-10 concentrations were observed in the different IL-10 genotypes (Fig. 2A). It was found that the GG/CC/CC genotype had significantly higher plasma concentrations of IL-10 (27.76±5.0 pg/ml) than the other genotypes. Other studies have documented that the IL-10 haplotype containing the G allele is associated with increased production of IL-10 (9,10), therefore, the plasma IL-10 concentrations of the haplotype based on the IL-10 producing capability were analyzed further. The haplotypes containing the G allele had significantly higher plasma IL-10 concentrations (24.33±5.7 pg/ml) compared with the ATA haplotype (19.88±4.7 pg/ml) (P=0.008; Fig. 2B).

## Discussion

IL-10 polymorphisms have been widely studied and are reported to be associated with certain other cancers and diseases; however, to the best of our knowledge, this is the first analysis of IL-10 polymorphisms and plasma IL-10 levels in patients with LSCC.

Immunity is mediated by Th1, Th2, Th3 and Treg responses, the balance of which affects the course of cytokine-mediated inflammation. IL-10 is a potent and pleiotropic cytokine that plays a crucial role in immunological and inflammatory responses, as it regulates B-cell proliferation and differentiation, and exhibits immunoregulatory activities and anti-inflammatory properties (18). Increased levels of IL-10 have been observed in patients with solid tumors, including oral squamous cell carcinoma, indicating that this cytokine has a significant role in carcinoma (19–21). It has been speculated that IL-10 contributes to the escape of tumor cells from immune surveillance and that it favors tumor growth.

The IL-10 gene is located on chromosome 1 (1q31–1q32). In recent decades, several SNPs have been investigated in the IL-10 gene region (17,22). It has been identified that there are three important polymorphisms in the 5-flanking region of the IL-10 gene at positions −1082, −819 and −592, which are associated with high transcriptional promoter activity (8). Numerous studies have reported associations between IL-10 polymorphisms and cancer susceptibility (23,24).

Different IL-10 promoter genotypes are associated with different levels of IL-10 production. Numerous studies have investigated the association between IL-10 promoter polymorphisms and the susceptibility to various types of cancers, but this association has not been examined in LSCC (12–16).

The purpose of the present study was to examine whether the IL-10 promoter polymorphisms and plasma IL-10 levels are associated with a risk for LSCC. The individuals under investigation were patients with LSCC or vocal leukoplakia, whose genotypes were compared with those of age- and gender-matched healthy controls.

In the study, it was demonstrated that the presence of the IL-10 −819 C, −592 C and −1082 G alleles were associated with an increased risk for LSCC development and vocal leukoplakia. The risk factors (OR) calculated in the present study indicated a 1.82-fold increased susceptibility to LSCC with the presence of AC at IL-10 −592 and −819 (P=0.024), while the OR for vocal leukoplakia was 1.93 (P=0.050). The relative risk (OR) of LSCC development for AG at −1082 heterozygotes was 2.20 (95% CI, 1.04–4.67; P=0.037; Table II), and the vocal leukoplakia OR was 2.14 (95% CI, 0.87–5.27; P=0.092; Table II). The −819 T/C SNP IL-10 polymorphism was in complete linkage disequilibrium with the −592 A/C SNP, which was in accordance with the observations of another study (18). Additionally, it was found that the OR for advanced LSCC was higher than for early-stage LSCC at the three positions, −1082, −819 and −592. Furthermore, the OR was significantly higher at −1082 GA/GG in the patients with lymph node metastasis, compared with those without (2.97 vs. 2.23; Table IV). Increased plasma IL-10 levels were detected in the patients with LSCC (P<0.01) and vocal leukoplakia (P<0.01) (Fig. 1A), while the level of plasma IL-10 was found to increase with the cancer staging. Furthermore, plasma IL-10 concentrations were significantly higher in the patients with lymph node metastasis (27.95±5.7 pg/ml) compared with those without metastasis (22.66±4.4 pg/ml) (P=0.01; Fig. 1C). The present findings indicate that the GG/CC/CC genotype is more common in patients with high IL-10 production, whereas AA/TT/AA is associated with low IL-10 production (Fig. 2A), which is consistent with the observations of McCarron *et al* (25). In addition, the haplotype containing the G allele was associated with higher plasma IL-10 concentrations (24.33±5.7 pg/ml) compared with the ATA haplotype (19.88±4.7 pg/ml) (P=0.008; Fig. 2B).

The association of cigarette smoking with an increased risk of LSCC and vocal leukoplakia was also found in the present study (LSCC: OR, 6.33; 95% CI, 3.7–10.8; P<0.01; vocal leukoplakia: OR, 4.73; 95% CI, 2.4–9.1; P<0.01). A similar association was identified for heavy alcohol consumption (LSCC: OR, 5.67; 95% CI, 3.3–9.9; P<0.01; vocal leukoplakia: OR, 5.70; 95% CI, 2.9–11.2; P<0.01). Thus, the present data indicates that environmental factors play a significant role in the pathogenesis of LSCC and vocal leukoplakia.

Certain limitations are present in the current study. First, other laryngeal carcinoma pathological types were not taken into account. Second, the sample size in the study was not large. Therefore, further studies should be conducted to further verify the results. However, the data further confirm that IL-10 levels are affected by numerous factors associated with the age and lifestyle of a patient, in accordance with the results of a previous study (26), although, the underlying mechanisms remain to be elucidated.

In conclusion, the present study indicates that the −592 A/C, −819 T/C and −1082 A/G polymorphisms of the IL-10 gene and increased plasma IL-10 levels are associated with an increased risk for LSCC and vocal leukoplakia. In addition, with the development of LSCC, higher IL-10 plasma levels and higher OR values of the −592 A/C, −819 T/C, −1082 A/G polymorphisms were found in the advanced LSCC patients and the patients with lymph node metastasis.

## Figures and Tables

**Table I:** Distribution of cases and controls according to selected sociodemographic characteristics.

Characteristics	Controls	LSCC	Vocal leukoplakia	OR (95% CI)^a^	P-value^a^	OR (95% CI)^b^	P-value^b^
Mean age ± SD, years	62.32±7.9	60.91±8.7	56.54±10.7				
Gender, n							
Female	5	4	2				
Male	114	142	59				
Smoking, n							
No	83	39	20	Reference		Reference	
Yes	36	107	41	6.33 (3.7–10.8)	<0.01	4.73 (2.4–9.1)	<0.01
Alcohol consumption, n							
No	95	60	25	Reference		Reference	
Yes	24	86	36	5.67 (3.3–9.9)	<0.01	5.70 (2.9–11.2)	<0.01
LSCC type, n (%)							
Glottic		98 (67.1)					
Supraglottic		47 (32.2)					
Subglottic		1 (0.7)					
LSCC stage, n (%)							
Advanced III + IV		50 (34.2)					
Early I + II		96 (65.8)					
Lymph node, n (%)							
N0		110 (75.3)					
N1+N2		36 (24.7)					

a OR, P-value calculated between LSCC and controls with SPSS;

b OR, P-value calculated between vocal-leukoplakia and controls with SPSS.

OR, odds ratio ; LSCC, laryngeal squamous cell carcinoma; CI, confidence interval; SD, standard deviation.

**Table II:** Association between IL-10 genotypes and development of LSCC and vocal leukoplakia.

Genotype	Controls (n=119)	LSCC (n=146)			Vocal leukoplakia (n=61)		
	
		n	OR (95% CI)	P-value	n	OR (95% CI)	P-value
IL-10 −592							
AA	64	63	Reference		23	Reference	
AC	39	70	1.82 (1.08–3.08)	0.024	27	1.93 (0.97–3.81)	0.050
CC	16	13	0.83 (0.37–1.86)	0.642	11	1.91 (0.78–4.72)	0.164
Alleles							
A	167	196	Reference		73	Reference	
C	71	96	1.15 (0.80–1.67)	0.453	49	1.58 (1.00–2.49)	0.049
IL-10 −819							
TT	64	63	Reference		23	Reference	
TC	39	70	1.82 (1.08–3.08)	0.024	27	1.93 (0.97–3.81)	0.050
CC	16	13	0.83 (0.37–1.86)	0.642	11	1.91 (0.78–4.72)	0.164
Alleles							
T	167	196	Reference		73	Reference	
C	71	96	1.15 (0.80–1.67)	0.453	49	1.58 (1.00–2.49)	0.049
IL-10 −1082							
AA	107	115	Reference		50	Reference	
AG	11	26	2.20 (1.04–4.67)	0.037	11	2.14 (0.87–5.27)	0.092
GG	1	5	4.65 (0.54–40.47)	0.127	0	--	
Alleles							
A	225	256	Reference		111	Reference	
G	13	36	2.43 (1.26–4.70)	0.007	11	1.72 (0.75–3.95)	0.201

IL-10, interleukin-10; LSCC, laryngeal squamous cell carcinoma; OR, odds ratio; CI, confidence interval.

**Table III:** Prevalence of IL-10 polymorphism in controls and patients in regard to early (I, II) and advanced (III, IV) cancer stages.

		Early (I+II)			Advanced (III+IV)		
			
Genotype	Controls, n	n	OR (95% CI)	P-value	n	OR (95% CI)	P-value
−592							
AA	64	42	Reference		21	Reference	
AC	39	46	1.80 (1.01–3.20)	0.046	24	1.88 (0.92–3.81)	0.080
CC	16	8	0.76 (0.30–1.94)	0.567	5	0.95 (0.31–2.92)	0.932
−819							
TT	64	42	Reference		21	Reference	
TC	39	46	1.80 (1.01–3.20)	0.046	24	1.88 (0.92–3.81)	0.080
CC	16	8	0.76 (0.30–1.94)	0.567	5	0.95 (0.31–2.92)	0.932
−1082							
AA	107	78	Reference		37	Reference	
AG	11	16	2.00 (0.88–4.54)	0.095	10	2.63 (1.03–6.69)	0.038
GG	1	2	2.74 (0.24–30.80)	0.396	3	8.68 (0.88–86.00)	0.060
AG+GG	12	18	2.06 (0.94–4.52)	0.068	13	3.13 (1.31–7.47)	0.008

IL-10, interleukin-10; OR, odds ratio; CI, confidence interval.

**Table IV:** Affect of IL-10 polymorphism on lymph node metastasis.

		Lymph node metastasis (−)			Lymph node metastasis (+)		
			
Genotype	Controls, n	n	OR (95% CI)	P-value	n	OR (95% CI)	P-value
−592							
AA	64	47	Reference		16	Reference	
AC+CC	55	63	1.56 (0.93–2.63)	0.094	20	1.46 (0.69–3.08)	0.326
−819							
TT	64	47	Reference		16	Reference	
TC+CC	55	63	1.56 (0.93–2.63)	0.094	20	1.46 (0.69–3.08)	0.326
−1082							
AA	107	88	Reference		27	Reference	
AG+GG	12	22	2.23 (1.05–4.76)	0.035	9	2.97 (1.14–7.78)	0.048

OR, odds ratio; CI, confidence interval.

## References

[1] Hashibe M, Brennan P, Benhamou S (2007). Alcohol drinking in never users of tobacco, cigarette smoking in never drinkers, and the risk of head and neck cancer: pooled analysis in the International Head and Neck Cancer Epidemiology Consortium. J Natl Cancer Inst.

[2] Moyer JS, Wolf GT, Bradford CR (2004). Current thoughts on the role of chemotherapy and radiation in advanced head and neck cancer. Curr Opin Otolaryngol Head Neck Surg.

[3] Boccia S, Cadoni G, Sayed-Tabatabaei FA (2008). CYP1A1, CYP2E1, GSTM1, GSTT1, EPHX1 exons 3 and 4, and NAT2 polymorphisms, smoking, consumption of alcohol and fruit and vegetables and risk of head and neck cancer. J Cancer Res Clin Oncol.

[4] de Waal Malefyt R, Abrams J, Bennett B, Figdor CG, de Vries JE (1991). Interleukin 10(IL-10) inhibits cytokine synthesis by human monocytes: an autoregulatory role of IL-10 produced by monocytes. J Exp Med.

[5] Moore KW, de Waal Malefyt R, Coffman RL, O’Garra A (2001). Interleukin-10 and the interleukin-10 receptor. Ann Rev Immunol.

[6] Akdis CA, Blaser K (2001). Mechanisms of interleukin-10-mediated immune suppression. Immunology.

[7] Wu MS, Huang SP, Chang YT (2002). Tumor necrosis factor-alpha and interleukin-10 promoter polymorphisms in Epstein-Barr virus-associated gastric carcinoma. J Infect Dis.

[8] Turner DM, Williams DM, Sankaran D, Lazarus M, Sinnott PJ, Hutchinson IV (1997). An investigation of polymorphism in the interleukin-10 gene promoter. Eur J Immunogenet.

[9] Crawley E, Kay R, Sillibourne J, Patel P, Hutchinson I, Woo P (1999). Polymorphic haplotypes of the interleukin-10 5′ flanking region determine variable interleukin-10 transcription and are associated with particular phenotypes of juvenile rheumatoid arthritis. Arthritis Rheum.

[10] Edwards-Smith CJ, Jonsson JR, Purdie DM, Bansal A, Shorthouse C, Powell EE (1999). Interleukin-10 promoter polymorphism predicts initial response of chronic hepatitis C to interferon alfa. Hepatology.

[11] Shimoyama T, Everett SM, Fukuda S, Axon AT, Dixon MF, Crabtree JE (2001). Influence of smoking and alcohol on gastric chemokine mRNA expression in patients with *Helicobacter pylori* infection. J Clin Pathol.

[12] Seifart C, Plagens A, Dempfle A (2005). TNF-alpha, TNF-beta, IL-6, and IL-10 polymorphisms in patients with lung cancer. Dis Markers.

[13] Erdogan M, Karadeniz M, Ozbek M, Ozgen AG, Berdeli A (2008). Interleukin-10 gene polymorphism in patients with papillary thyroid cancer in Turkish population. J Endocrinol Invest.

[14] Kesarwani P, Ahirwar DK, Mandhani A (2009). IL-10 −1082 G>A: a risk for prostate cancer but may be protective against progression of prostate cancer in North Indian cohort. World J Urol.

[15] Matsumoto K, Oki A, Satoh T (2010). Interleukin-10 −1082 gene polymorphism and susceptibility to cervical cancer among Japanese women. Jpn J Clin Oncol.

[16] Zhou Y, Hu W, Zhuang W, Wu X (2011). Interleukin-10 −1082 promoter polymorphism and gastric cancer risk in a Chinese Han population. Mol Cell Biochem.

[17] Wu MS, Wu CY, Chen CJ, Lin MT, Shun CT, Lin JT (2003). Interleukin-10 genotypes associate with the risk of gastric carcinoma in Taiwanese Chinese. Int J Cancer.

[18] Gibson AW, Edberg JC, Wu J, Westendorp RG, Huizinga TW, Kimberly RP (2001). Novel single nucleotide polymorphisms in the distal IL-10 promoter affect IL-10 production and enhance the risk of systemic lupus erythematosus. J Immunol.

[19] Fortis C, Foppoli M, Gianotti L (1996). Increased interleukin-10 serum levels in patients with solid tumours. Cancer Lett.

[20] Karcher J, Reisser C, Daniel V, Herold-Mende C (1999). Cytokine expression of transforming growth factor-beta2 and interleukin-10 in squamous cell carcinomas of the head and neck. Comparison of tissue expression and serum levels. HNO.

[21] Fujieda S, Sunaga H, Tsuzuki H, Fan GK, Saito H (1999). IL-10 expression is associated with the expression of platelet-derived endothelial cell growth factor and prognosis in oral and oropharyngeal carcinoma. Cancer Lett.

[22] Zambon CF, Basso D, Navaglia F (2005). Pro- and anti-inflammatory cytokines gene polymorphisms and *Helicobacter pylori* infection: interactions influence outcome. Cytokine.

[23] Rad R, Dossumbekova A, Neu B (2004). Cytokine gene polymorphisms influence mucosal cytokine expression, gastric inflammation, and host specific colonisation during *Helicobacter pylori* infection. Gut.

[24] Suzuki S, Muroishi Y, Nakanishi I, Oda Y (2004). Relationship between genetic polymorphisms of drug-metabolizing enzymes (CYP1A1, CYP2E1, GSTM1, and NAT2), drinking habits, histological subtypes, and p53 gene point mutations in Japanese patients with gastric cancer. J Gastroenterol.

[25] McCarron SL, Edwards S, Evans PR, Gibbs R, Dearnaley DP, Dowe A, Southgate C, Easton DF, Eeles RA, Howell WM (2002). Influence of cytokine gene polymorphisms on the development of prostate cancer. Cancer Res.

[26] Gravitt PE, Hildesheim A, Herrero R (2003). Correlates of IL-10 and IL-12 concentrations in cervical secretions. J Clin Immunol.

